# Supplementary material for: Cell-Free DNA Hypermethylation in Patients with Acute Pancreatitis
Source: Int J Mol Sci. 2025 Nov 6;26(21):10792. doi: 10.3390/ijms262110792 (PMC12609792; doi:10.3390/ijms262110792)
Supplement: Supplementary file 1 [file ijms-26-10792-s001.zip › Supplementary table S1.pdf]

Supplementary table S1- Frequencies of each Gene in Different Time-Points in both Patients and Healthy Controls

	**AP-T0** (n=61)	**AP-T6W** (n=43)	**AP-T6M** (n=33)	**AP-T8Y** (n=28)	**Healthy controls** (n=70)
ALX4	1 (2%)	3 (7%)	0 (0%)	2 (7%)	1 (1%)
APC	**42** (69%)	15 (35%)	17 (52%)	16 (57%)	25 (36%)
BMP3	**13** (21%)	**5** (12%)	**6** (18%)	0 (0%)	1 (1%)
BNC1	2 (3%)	3 (7%)	3 (9%)	0 (0%)	3 (4%)
BRCA1	**24** (39%)	**10** (23%)	**8** (24%)	2 (7%)	5 (7%)
CDKN2B	**9** (15%)	**4** (9%)	**3** (9%)	0 (0%)	0 (0%)
ESR1	**58** (95%)	**30** (70%)	**21** (64%)	12 (43%)	25 (36%)
EYA2	**12** (20%)	**10** (23%)	**11** (33%)	0 (0%)	0 (0%)
HIC1	6 (10%)	1 (2%)	1 (3%)	1 (4%)	2 (3%)
MEST1v2	**51** (84%)	**29** (67%)	**21** (64%)	6 (21%)	22 (31%)
MGMT	1 (2%)	0 (0%)	0 (0%)	0 (0%)	1 (1%)
Neurog1	**15** (25%)	5 (12%)	5 (15%)	2 (7%)	3 (4%)
NPTX2	**46** (75%)	**28** (65%)	**14** (42%)	3 (11%)	9 (13%)
p16	**11** (18%)	3 (7%)	3 (9%)	0 (0%)	2 (3%)
RARB	**51** (84%)	**30** (70%)	**22** (67%)	**2** (7%)	17 (24%)
RASSF1A	**32** (52%)	8 (19%)	8 (24%)	**15** (54%)	20 (29%)
Sept9v2	3 (5%)	1 (2%)	1 (3%)	0 (0%)	0 (0%)
SFRP1	5 (8%)	**0** (0%)	**0** (0%)	7 (25%)	15 (21%)
SFRP2	8 (13%)	5 (12%)	0 (0%)	0 (0%)	4 (6%)
Sst1	21 (34%)	18 (42%)	9 (27%)	4 (14%)	20 (29%)
TFPI2	1 (2%)	1 (2%)	0 (0%)	0 (0%)	1 (1%)
Tac1	31 (51%)	16 (37%)	11 (33%)	7 (25%)	28 (40%)
WNT5A	**9** (15%)	**1** (2%)	**0** (0%)	11 (39%)	27 (39%)

Demonstrates the frequencies of individual hypermethylated genes in AP patients and healthy controls at different time points. Those highlighted in bold and marked in green are statistically significantly higher from the healthy controls. Those marked in orange are statistically significantly lower from the healthy controls (using Fisher's exact test, P-value < 0.05).
