# Supplementary material for: Cell-Free DNA Hypermethylation in Patients with Acute Pancreatitis
Source: Int J Mol Sci. 2025 Nov 6;26(21):10792. doi: 10.3390/ijms262110792 (PMC12609792; doi:10.3390/ijms262110792)
Supplement: Supplementary file 1 [file ijms-26-10792-s001.zip › Supplementary table S2.pdf]

Supplementary table S2 – Associations Between Hypermethylation of Individual Genes in the 23-Gene Panel and Proxy-markers of Acute Pancreatitis Severity

Association Between Hypermethylation of Individual Genes at T0 and Length of Hospital Stay

	**Non-hypermethylated group** Median (IQR)	**Hypermethylated group** Median (IQR)	**p-value**
ALX4T0	6.0 (4–8)	5.0 (5–5)	0.606
APCT0	6.0 (4.5–8)	6.0 (4.25–8)	0.561
BMP3T0	6.0 (4–8)	7.0 (5–8)	0.429
BNC1T0	6.0 (4–8)	5.0 (5–5)	0.450
BRCA1T0	5.0 (4–7)	8.0 (5–11)	0.005
CDKN2BT0	6.0 (4–8)	6.0 (5–8)	0.984
ESR1T0	10.0 (7–10.5)	6.0 (4.25–8)	0.501
EYA2T0	6.0 (4–8)	5.5 (4.75–7.25)	0.694
HIC1T0	6.0 (4–8)	10.5 (8.5–13.25)	**0.007**
MEST1v2T0	6.0 (5–10)	6.0 (4–8)	0.644
MGMTT0	6.0 (4–8)	5.0 (5–5)	0.606
Neurog1T0	6.0 (4.5–7)	6.5 (4.25–8.75)	0.403
NPTX2T0	6.0 (4–8)	7.0 (5–10)	0.190
p16T0	6.0 (4–8)	7.0 (5–7.5)	0.741
RARBT0	5.5 (4–6)	7.0 (5–8.5)	0.105
RASSF1AT0	5.0 (4–7)	7.0 (5–9.25)	0.018
Sept9v2T0	6.0 (5–8)	4.0 (3–51.5)	0.512
SFRP1T0	6.0 (4–8)	8.0 (7–14)	0.160
SFRP2T0	7.0 (5–8)	5.0 (4–5.25)	0.075
Sst1T0	6.0 (4–8.5)	5.0 (5–8)	0.872
Tac1T0	6.0 (4–7.75)	6.0 (5–8)	0.395
TFPI2T0	6.0 (4–8)	5.0 (5–5)	0.606
WNT5AT0	6.0 (4–8)	7.0 (5–10)	0.854

Table 1: The association between hypermethylation of individual genes at T0 and length of hospital stay (in days). For each gene, patients were grouped based on the presence or absence of hypermethylation T0, and length of hospital stay were compared using the Wilcoxon rank-sum test. Values are shown as median and interquartile range (IQR). Bold p-values indicate statistically significant differences at $p < 0.05$.

Association Between Hypermethylation of Individual Genes at T0 and The Maximum CRP During Admission

	**Non-hypermethylated group** Median (IQR)	**Hypermethylated group** Median (IQR)	**p-value**
ALX4	156 (49–241.25)	147 (147–147)	0.977
APC	46 (14.55–193)	173 (97.75–254)	**0.008**
BMP3	151.5 (46–222.25)	152 (100–268)	0.418
BNC1	160 (48–244.5)	111 (93–129)	0.613
BRCA1	123 (46–212)	194.5 (72.25–330.25)	**0.075**
CDKN2B	156 (60.5–222.25)	147 (39–337)	0.919
ESR1	238 (134.5–290.5)	149.5 (52–228.75)	0.537
EYA2	152 (46–232)	128.5 (80.75–255.25)	0.885
HIC1	146 (48–215.5)	293 (224–389.75)	**0.034**
MEST1v2	168.5 (57.75–212)	147 (52–244.5)	0.868
MGMT	149.5 (49–233.5)	268 (268–268)	0.307
Neurog1	81 (26–144.5)	193 (76.25–264.75)	**0.016**
NPTX2	153 (47–228.75)	152 (92.5–313.5)	0.393
p16	149 (47–236.5)	208 (103.5–243.5)	0.459
RARB	63 (20.5–129.75)	169 (69.5–261.5)	**0.019**
RASSF1A	117 (31–232)	166 (80.75–263)	0.182
Sept9v2	156 (59.5–236.5)	40 (21–254.5)	0.605
SFRP1	156 (56–241.25)	143 (23–169)	0.608
SFRP2	147 (58–219)	231.5 (44.75–275.75)	0.608
Sst1	161.5 (42.25–222.25)	147 (75–251)	0.514
Tac1	110 (29.5–214.25)	163 (87.5–283.5)	0.113
TFPI2	156 (49–241.25)	147 (147–147)	0.977
WNT5A	149.5 (49–233.5)	181 (75–328)	0.597

Table 2: The association between hypermethylation of individual genes at T0 and maximum C-reactive protein (CRP) at hospital admission. For each gene, patients were grouped based on the presence or absence of hypermethylation, and CRP levels were compared using the Wilcoxon rank-sum test. Values are shown as median and interquartile range (IQR). Bold p-values indicate statistically significant differences at $p < 0.05$.

Association Between Hypermethylation of Individual Genes at T0 and the Presence of Complications During Admission

	**Non-hypermethylated group** n (%)	**Hypermethylated group** n (%)	**p-value**
ALX4	15 (25.0%)	1 (100.0%)	0.262
APC	5 (26.3%)	11 (26.2%)	1.000
BMP3	10 (20.8%)	6 (46.2%)	0.083
BNC1	15 (25.4%)	1 (50.0%)	0.459
BRCA1	9 (24.3%)	7 (29.2%)	0.769
CDKN2B	13 (25.0%)	3 (33.3%)	0.686
ESR1	2 (66.7%)	14 (24.1%)	0.166
EYA2	13 (26.5%)	3 (25.0%)	1.000
HIC1	14 (25.5%)	2 (33.3%)	0.648
MEST1v2	4 (40.0%)	12 (23.5%)	0.431
MGMT	16 (26.7%)	0 (0.0%)	1.000
Neurog1	2 (13.3%)	14 (30.4%)	0.312
NPTX2	12 (26.1%)	4 (26.7%)	1.000
p16	11 (22.0%)	5 (45.5%)	0.137
RARB	1 (10.0%)	15 (29.4%)	0.267
RASSF1A	8 (27.6%)	8 (25.0%)	1.000
Sept9v2	15 (25.9%)	1 (33.3%)	1.000
SFRP1	16 (28.6%)	0 (0.0%)	0.313
SFRP2	12 (22.6%)	4 (50.0%)	0.189
Sst1	8 (20.0%)	8 (38.1%)	0.141
Tac1	10 (33.3%)	6 (19.4%)	0.255
TFPI2	15 (25.0%)	1 (100.0%)	0.262
WNT5A	13 (25.0%)	3 (33.3%)	0.686

Table 3: The number and percentage of patients with at least one complication during admission, stratified by the methylation status of 23 individual genes. For each gene, the number and proportion of patients experiencing complications are shown separately for the non-hypermethylated and hypermethylated groups. Associations were tested using Fisher's exact test.

Association Between Hypermethylation of Individual Genes at T0 and The Maximum creatinine During Admission

	**Non-hypermethylated group** Median (IQR)	**Hypermethylated group** Median (IQR)	**p-value**
ALX4	76.5 (61–92.5)	40.0 (40–40)	0.094
APC	64.0 (59.5–80.5)	80.0 (61.5–99.25)	0.125
BMP3	76.5 (60.75–92.5)	69.0 (61–89)	0.711
BNC1	77.0 (61–94)	50.0 (45–55)	0.060
BRCA1	64.0 (60–86)	84.0 (67.5–116)	**0.035**
CDKN2B	77.0 (60.75–92.5)	69.0 (62–82)	0.569
ESR1	72.0 (64.5–77)	76.5 (61–95.5)	0.526
EYA2	77.0 (61–97)	72.5 (59.75–87.25)	0.690
HIC1	77.0 (61–94)	66.5 (61–75)	0.594
MEST1v2	77.0 (66–89.75)	75.0 (60–93)	0.508
MGMT	75.5 (60.75–91)	91.0 (91–91)	0.410
Neurog1	64.0 (61–87)	77.0 (60.25–97)	0.431
NPTX2	73.5 (61–88.75)	82.0 (62–129.5)	0.299
p16	75.5 (61–95.5)	77.0 (58.5–86.5)	0.561
RARB	62.5 (58–80.75)	77.0 (61–98.5)	0.114
RASSF1A	64.0 (60–82)	86.0 (63.25–105.25)	**0.041**
Sept9v2	75.5 (61–89)	101.0 (79.5–133.5)	0.395
SFRP1	75.5 (60.75–92.5)	77.0 (71–86)	0.895
SFRP2	76.0 (60–97)	77.0 (63.25–88)	0.716
Sst1	76.0 (60.75–97)	76.0 (61–89)	0.820
Tac1	64.0 (60–85.75)	77.0 (64–98.5)	0.164
TFPI2	76.5 (61–92.5)	40.0 (40–40)	0.094
WNT5A	75.5 (61–89)	82.0 (60–112)	0.839

Table 4: The association between hypermethylation of individual genes at T0 and maximum creatinine concentration at hospital admission. For each gene, patients were grouped based on the presence or absence of hypermethylation, and creatinine levels were compared using the Wilcoxon rank-sum test. Values are shown as median and interquartile range (IQR). Bold p-values indicate statistically significant differences at $p < 0.05$.

Association Between Hypermethylation of Individual Genes at T0 and The Maximum Leucocyte Count During Admission

	**Non-hypermethylated group** Median (IQR)	**Hypermethylated group** Median (IQR)	**p-value**
ALX4	11.65 (8.18–16.90)	25.40 (25.4–25.4)	0.105
APC	10.30 (7.15–12.10)	12.65 (8.73–18.45)	0.091
BMP3	11.05 (8.18–16.28)	13.40 (9.60–21.40)	0.211
BNC1	11.80 (8.15–17.00)	17.65 (13.78–21.53)	0.395
BRCA1	10.80 (8.10–14.50)	14.55 (9.35–19.53)	0.070
CDKN2B	11.05 (8.10–16.28)	19.60 (12.30–22.10)	**0.028**
ESR1	16.10 (13.65–19.10)	11.65 (8.13–17.10)	0.264
EYA2	11.50 (8.20–16.10)	15.15 (8.10–21.80)	0.297
HIC1	10.90 (8.10–16.35)	19.85 (16.65–23.58)	**0.005**
MEST1v2	11.85 (8.70–16.95)	11.50 (8.15–17.00)	0.907
MGMT	11.80 (8.18–17.48)	10.20 (10.2–10.2)	0.755
Neurog1	10.90 (7.70–12.25)	12.10 (8.73–18.50)	0.097
NPTX2	11.35 (8.05–15.88)	15.90 (9.10–21.20)	0.106
p16	11.85 (8.10–17.10)	10.90 (9.10–16.50)	0.714
RARB	7.95 (7.25–15.03)	11.90 (8.85–18.50)	0.083
RASSF1A	10.80 (8.20–16.10)	12.10 (8.08–18.50)	0.554
Sept9v2	11.80 (8.20–18.03)	8.60 (6.55–10.90)	0.257
SFRP1	11.80 (8.20–18.35)	10.90 (7.20–14.50)	0.462
SFRP2	11.80 (8.20–17.20)	11.00 (7.68–16.15)	0.693
Sst1	11.65 (7.70–16.80)	12.30 (9.90–19.60)	0.121
Tac1	11.35 (8.10–15.03)	12.70 (9.50–18.75)	0.197
TFPI2	11.65 (8.18–16.90)	25.40 (25.4–25.4)	0.105
WNT5A	11.65 (8.18–16.80)	12.60 (9.60–19.60)	0.542

Table 5: The association between hypermethylation of individual genes at T0 and maximum leucocyte count level at hospital admission. For each gene, patients were grouped based on the presence or absence of hypermethylation, and leucocyte count levels were compared using the Wilcoxon rank-sum test. Values are shown as median and interquartile range (IQR). Bold p-values indicate statistically significant differences at $p < 0.05$.
